# Inclusivity for children with autism spectrum disorders: Parents’ reflections of the school learning environment versus home learning during COVID-19

**DOI:** 10.1080/20473869.2021.1975253

**Published:** 2021-09-20

**Authors:** C. Hill, S. Keville, A. K. Ludlow

**Affiliations:** Department of Psychology, Sport and Geography, University of Hertfordshire, College Lane, Hatfield, Herts, Al10 9ab, United Kingdom

**Keywords:** autism spectrum disorder, health issues, inclusivity, learning environments, COVID-19, transition, home-learning, school-learning

## Abstract

Whilst attendance in mainstream school helps encourage inclusivity, these environments are recognised as being particularly challenging for young people with an autism spectrum disorder (ASD). The COVID-19 pandemic brought a novel transition as young people moved from school to home-learning. This study compared the experiences of parents of children with ASD and co-occurring health difficulties of school-learning environments with their home-learning environments during COVID-19 lockdown.

An interpretative phenomenological analysis was conducted on semi-structured interviews from six parents of children (aged 8-15 years old) with ASD. Four superordinate themes were generated: Interactions between Health, ASD and Learning Environments; School Support and Managing Health Needs; Seeking Solutions; and Learning from COVID-19 Lockdown.

The study highlighted the impact of ASD and co-occurring health difficulties on learning where parents found ways to provide positive home-learning environments which could be used and/or transferred back into school environments. These results hold real-world implications where educators could treat sensory and ASD friendly environments as standard and include genuine adjustments for children with ASD and additional needs. A flexible home-learning approach using parental knowledge around environmental adjustments that support learning, wellbeing and a sense of inclusion should be prioritised for children’s overall development and wellbeing in these unprecedented times, and beyond.

## Introduction

Autism spectrum disorder (ASD) is a neurodevelopmental disorder involving difficulties with social communication and repetitive behaviours (American Psychiatric Association, 2013). In schools in England, the standardised prevalence is around 1.76% of children (Roman-Urrestarazu *et al.*, 2021). The rate of co-occurring issues is high (Matson and Nebel-Schwalm, 2007) with 70% having at least one co-occurring physical, psychological or neurodevelopmental diagnosis, and 41% having two or more (Simonoff *et al.*, 2008). The most common are social anxiety disorder (28%; Bejerot *et al.*, 2014); attention deficit hyperactivity disorder (ADHD)(59%; Stevens, Peng and Barnard-Brak, 2016); at least one anxiety disorder (29.6%; van Steensel *et al.*, 2011); and gastrointestinal problems (9-91%; Mannion and Leader, 2013); including abdominal pain (2-41%), constipation (6-45%), and diarrhoea (3-77%; Coury *et al.*, 2012).

Co-occurring issues may impact on the experience of learning and learning environments for those with ASD. For example, compared to those with ASD alone, individuals with co-occurring ADHD are more impaired in their daily living skills, have lower IQ scores, higher cognitive and social difficulties, higher anxiety and perfectionism levels (Craig *et al.*, 2015); higher rates of hyperactivity and attention difficulties (Yerys *et al.*, 2009); and more conduct-related issues than peers (Jang *et al.*, 2013). Perhaps this is compounded by the lack of understanding and interventions for co-occurring issues in ASD; for example, it has been suggested that ADHD is a pharmaco-responsive psychiatric condition, yet young people with ASD and co-occurring ADHD may receive no treatment, potentially impacting on their academic performance (Joshi *et al.*, 2017).

Despite global initiatives for inclusivity in education, this still appears to be elusive. For example, this has been noted as a specific issue for ASD in China (Zhao *et al.*, 2021). Furthermore, educational outcomes in school environments for those with ASD are lower than expected (Tsatsanis, 2003; Goodall, 2019), even though there is an assumption that academically able young people with ASD should be able to cope in mainstream classrooms (Humphrey and Lewis, 2008; Goodall, 2019). Perhaps such assumptions also drive those with ASD to use emotionally and cognitively demanding strategies, such as masking of their ASD symptoms, to avoid stigma (Pearson and Rose, 2021). Yet, masking may also relate to negative outcomes on mental health, burnout and suicidality in ASD (Pearson and Rose, 2021). Given young people with ASD and a co-occurring issue are at greater risk of victimisation compared to peers (Sterzing et al., 2012), adaptation within learning environments seems crucial for these children.

Environments children learn in include school or home settings and represent the social, psychological and physical environment for learning (Cleveland and Fisher, 2014). It is within this context where student attainment in learning is determined, often by the amount of time students are actively engaged in learning, known as academic engaged time (AET); AET strongly predicts attainment, and is influenced by student motivation (Gettinger and Walter, 2012). Whilst AET is important, less than half of designated lesson time is spent actively engaged in learning, and only around 38% of the traditional school day is dedicated to lessons (Rangel and Berliner, 2007). Thus, a large proportion of time involves less structured learning and social activities; arguably the areas children with ASD have deficits in (APA, 2013). Indeed, as children with ASD progress through school, participation in school activities reduces, alongside a more general reduction in unstructured and organised physical activities (Simpson *et al.*, 2019). The gap is such that many parents of children with special needs or disabilities choose to home educate due to the deleterious impact of poor school-learning environments on their child (Bauman, 2002; Isenberg, 2007; Kendall and Taylor, 2016). Indeed, home-educated children with learning disabilities have higher AET and make larger gains in their reading, math and written language compared to higher non-compliance and disruptiveness in tasks for those in school settings; the reading ability of those in schools decreased over the same time period as those making gains at home (Duvall *et al.*, 1997). Similarly, there is higher academic achievement for those in structured home-learning compared to traditional schooling (Martin-Chang *et al.*, 2011).

It is important to understand why a home-learning environment may hold some advantages for some children with additional needs and more systematically utilise these strategies in school environments; this information could enhance flexibility in education provision for young people with ASD (Lawrence, 2012). For example, given school-based transitions are a known source of distress in children with ASD (Makin *et al.*, 2017), understanding how children managed recent transitions into home-learning environments may facilitate the generalisation of these skills into the school environment, particularly during times of transition. This seems timely given some successful generalisation of learning can occur for children with ASD across settings, people, and/or activities (Carruthers *et al.*, 2020).

In the United Kingdom (UK), since March 2020, national lockdown to manage the pandemic resulted in transitions to home-learning for all school-aged children (Ofsted, 2020). The concern for its impact resulted in the Ofsted chief inspector stating school closures should now be kept to the absolute minimum to avoid disrupting children's learning and wider development (Hope and Bird, 2021). Indeed, the Ofsted chief inspector reported that some younger children had forgotten how to hold a pencil, or use a knife and fork, and some had regressed in basic language skills during lockdown, with additional increases in eating disorders, self-harm, and anti-social behaviour in older children (Hope and Bird, 2021). Yet this is for the majority, and often typically developing children.

Due to the ongoing COVID-19 pandemic and nationwide lockdown, unprecedented events have now meant most young people in the UK have experienced home-learning, which had not previously been a legitimate option. Consequently, this provided a unique opportunity to explore contrasts and adaptations between school and home-learning environments for those with ASD. Research is minimal and mainly on accounts from small samples in other countries (for example, Zimabwe (Majoko and Dudu, 2020); Philippines (Cahapay 2020). To our knowledge this is the first study to explore richly and directly the lived experience of learning environments in the UK from the perspective of parents of young people with ASD.

## Materials and methods

### Research design

A qualitative design was chosen due to its ideographic approach and ecological validity; thus, interpretative phenomenological analysis was utilised to explore the lived experiences of parents of young people with ASD and additional health difficulties, and how this was navigated within different learning environments (Smith *et al.*, 2009).

### Participants

For recruitment, advertisements were placed with local online support groups and snowballing methods used. Six parents responded and all were recruited as they met the inclusion criteria of having a child with a diagnosis of ASD, co-occurring health issues and attendance within secondary mainstream education in the UK.

### Instrument

Demographic data were collected (Table 1), and experiences explored through semi-structured interviews.

**Table 2.** Superordinate and Subordinate Themes.

Superordinate theme	Subordinate themes
Interactions between Health, ASD and Learning Environments	The Impact of Health Issues on Learning
	Mismatch of ASD Features and School Environments
School Support and Managing Health Needs	Reasonable Adjustments and Experiences of Support
	Masking Additional Needs
Seeking Solutions	Parental Learning Interventions
	Fostering Communication and Relationships
Learning from COVID-19 Lockdown	Discovering the Benefits of Home-Learning
	Transitions during a Pandemic

The interview schedule was developed through gaps and issues raised in the literature and was refined by the research team following consultation with a parent with lived experience. Questions included:

- In what ways does autism spectrum disorder (ASD)/health issues impact on your child, their relationships with others and your child’s learning?
- How has the COVID-19 pandemic and lockdown impacted on your child and your child’s learning at present?
- How are you managing your child’s learning at home compared to learning in a school setting?
- When you look back to when your child was able to attend school, was there anything you found helpful or unhelpful at the time to manage your child’s health alongside school attendance?

### Data collection

The first author conducted all interviews in July 2020 during the first UK lockdown which began in March 2020. Due to the social distancing measures, interviews were conducted through a video conferencing online platform, and lasted between 68-92 min (*M* = 78.7, SD = 13.93). Following the interview, participants were given the opportunity to share further information, thanked for their time and were given a debrief sheet. Video recordings were made of each interview and were transcribed verbatim by the lead author.

### Ethical considerations

Approval was given by the institution’s Ethics Committee (protocol number: aLMS/PGT/UH/04165(2)). Confidentiality was ensured by the researchers throughout the study. Consent was sought from the participants who were made aware of interviews being recorded, stored, deleted on transcription and anonymised data used in a publication. During transcription, identifying details were removed and pseudonyms applied. Following the interview participants were provided with a debrief sheet including supportive websites.

### Data analysis

Interpretative phenomenological analysis (IPA) was used to interpret the participants’ subjective experience (Smith *et al.*, 2009). The lead author read and listened several times to the transcript and recordings to ensure familiarity before analysis, noting emerging themes, comments and interpretations; interrater reliability was conducted with the second author.

Emerging themes were collated across transcripts and analysed for similarities and divergencies, creating superordinate and subordinate themes across the data. These were checked against the transcripts to ensure participants words were represented. Interrater reliability was again conducted with the second author to refine the themes. To ensure rigour and credibility the final themes table with supporting quotes was developed with the second author. The finalised table was shared with all participants; two responded, and wording suggestions made by one participant were incorporated (Tracy, 2010).

## Results

Analysis of six semi-structured interviews resulted in the following subordinate and superordinate themes (Table 2).

### Interactions between health, ASD and learning environments

All parents discussed different aspects within different learning environments which interacted positively or negatively with their child’s health or ASD and impacted their learning. There was often a mismatch between aspects of the school environment and their child’s ASD symptoms.

#### The impact of health issues on learning

All parents spoke about the impact their child’s ASD and/or health difficulties had on their experience within the learning environment. For example, Allison spoke about her child’s anxieties around sickness:

…If a child put their hand up to say they didn’t feel very well in primary school, he would beat Usain Bolt off the starting blocks and be out of the classroom before the teachers even realised that somebody put their hand up. So if somebody sneezed, somebody coughed, he’d be gone; he was a complete flight risk.

The fear of catching an illness from another child triggered such an extreme desperation to escape classroom learning that Allison’s son became faster than the fastest runner in the world (‘Usain Bolt’). Lydia, a mother of a child with dyspraxia, also spoke of the impact of her child’s condition on his learning:

He gets tired in his hands and starts writing really bad…So when he starts writing bad, then he kind of loses all confidence, especially if he sat next to someone that writes really well.

Clearly handwriting, as an essential learned skill, was physically difficult for Lydia’s child, placing him at a physical disadvantage; ultimately when his writing was affected or he was reminded of his struggle compared to others, this took away his ‘confidence’ rather than built it.

When schools supported parental decisions to prioritise wellbeing over grades, parents and their children seemed to benefit from this, as Ryan stated when his son’s school allowed him to drop subjects so that he could concentrate on core ones:

What’s been quite good…because we've had that repetitive learning…his development in those subjects have actually gone up quite a lot.

#### Mismatch between ASD features and school environments

For those parents who felt unsupported by schools, there seemed to be a mismatch between their child’s ASD features/additional health needs and the school-learning environment. For example, the power of words between the communications of a teacher and Lydia’s child could have implications that only time, and re-building confidence, could redress:

His teacher said…his writing is ‘letting him down’…I had to sit there and say to her…’You have to watch your words with him, because you've said that…he's now shut down and he's not gonna do any work…until he's got that confidence back up, because…in his eyes, he's disappointed you.’

Rachel’s child had ASD and sensory issues which meant he ‘gets so distracted by anything that’s going on around him’. She went on to say:

He's got extremely sensitive hearing…So even somebody tapping their pencil bothers him and he can't focus on what he’s supposed to be focusing on, so he can't filter out….So that was a massive barrier to his learning.

The extremity of the ‘barriers’ to learn were evocatively emphasised by Rachel’s term ‘massive’ making it seem almost impossible for her son to experience a sense of comfort in learning within the busy school environment, let alone, to feel included within it.

### School support and managing health needs

Parents discussed the management of their children’s ASD and health difficulties and the impacts adjustments had. Parents noted their child’s tendency to mask their additional needs and its negative impact as their child felt unable to utilise the supports put in place.

#### Reasonable adjustments and experiences of support

Depending on individual experiences, all parents talked about the support their child received from schools. Three parents shared positive stories, such as Ryan who highlighted beneficial supports being put in place by staff:

…There’ll be times where his brain would just be flipping at a million miles an hour. So the teachers would rather that he left for five minutes and came back and they get 55 minutes of concentrated work off him, rather than lose 30 minutes where his brain is just all over the place.

The ability to take breaks and self-manage stimulation seemed to be a productive strategy which enhanced learning for Ryan’s son.

The level of support seemed dependent on the willingness of each school to support children’s learning needs. For example, Rachel embodied a mixed experience when she spoke about the positive impact of moving schools and the importance of finding the correct establishment for her child:

He's been happy and they seem to have been doing their job and they seem to be supporting him. So it's been a much better relationship. And it's reflected in the way that he is in school, because he's happy. And he hasn't been happy at school for three years…it kind of shows you that it was just the wrong place.

An appropriate environment with supportive educators seemed pivotal to the happiness of Rachel’s child; if a better place had not been found, one can only imagine the emotional and educational impact of remaining in ‘the wrong place’ beyond the three years her child had already endured.

For others, the experience of support was not always consistent as their children moved through school, or schools. Richard spoke about school’s ability to find solutions, yet, inflexibility in adapting this over time:

The solution is good in the time, but you've got to be able to adapt over time and the schools are very rigid, black and white organisations that aren't able to do that…They will put a plaster on a cut, but it can go septic under that plaster and they won’t realise.

Four parents spoke about reasonable adjustments to best support their child. For example, Allison stated: ‘So they're really good at…not trying to single him out, whilst trying to meet his needs’. In contrast, parents such as Richard spoke more negatively about school attitudes towards adjustments:

He’s being punished for things he can't help, so he fidgets that's branded as naughty, so we must punish him…They keep banging on about reasonable adjustments…but it's all box ticking, it's not real. It's not genuinely tailored to help your child, it's just mitigated against being accused of not doing their job properly.

Again, parents were able to articulate a school-based response to management. For Richard, the language used (‘box-ticking’, ‘mitigated against being accused’) made adjustments seem disingenuous, negating their purpose. Difficulties with the school were reiterated by Rachel:

And even after he was diagnosed, I had huge battles with school to get them to do anything that was reasonable adjustments for him. So school was really difficult.

#### Masking additional needs

Further to the struggle of parents accessing appropriate recognition of their child’s needs, four parents spoke about their child masking their additional needs and unwillingness to use supports, as illustrated by Richard:

Henry’s got…a traffic light system in his notebook, so if he's starting to bubble over, he could turn it to yellow and then to red…There is no way Henry’s showing them different colours, because his friends will see.

Drawing attention to their difference or need seemed to underpin this unwillingness, evoked through the phrase ‘no way’ and a sense of being fearful that his ‘friends will see’, perhaps indicating embarrassment if he used his supports. Extending this, Allison spoke about her child’s worry about being ‘bullied’ due to his cerebral palsy:

He's worried that he'll get bullied because of his involuntary movements, because people think it's a bit weird and bit odd. So he tries really hard to contain it at school, I suppose it's masking and then comes home. And it's shocking…It's just panic attack after panic attack and he can't feel his legs and he can't walk…his lips go blue and he gets himself in an absolute state.

Again, the worry was so great that the stress of ‘masking’ at school had an enormous impact on his health and wellbeing. Richard spoke about the complexity of having ASD:

So the bell that’s total neuro-overload…He's a bit worried about asking for help, because his friends might think he's not terribly good or clever…So now he's sitting there not understanding, it's all going over his head. So he's gonna to try and distract, rather than letting them know that he's struggling, he'll do something stupid to mask the fact that he's now out of his depth and he's missing out on the activity.

The totality of the overwhelmingness of expectations and rules within the school environment for a child with ASD was articulated by Richard’s term ‘total neuro-overload’. One can only imagine the experience of his son feeling ‘out of his depth’ within the school environment, impacting on his behaviour and making him spiral further away from comprehension and understanding. It was not always clear why children masked their difficulties, and this may be a factor influencing a lack of action by others as they focussed on the observable ‘stupid’ behaviours.

### Seeking solutions

All parents continued to seek solutions to foster inclusivity for their child’s overall development which could accommodate and adapt to their additional needs. It was through this that they found alternative ways to support learning and acted like a communication bridge between their child and the school.

#### Parental learning interventions

All parents spoke about strategies they used to assist their child’s adaptation to changes that had the potential to enhance their wellbeing or learning. For example, Richard would maintain a learning routine in the holidays to help his child retain knowledge:

Even through the summer holidays, we've been around for him to have his Maths lessons, because we're a bit conscious if you give him a six-week break, he's going to forget everything. We don't want that coming into the final year of GCSEs.

On top of parental interventions to support learning, parents such as Sarah spoke about their involvement in school-based interventions:

The school trip, they went away for a week to the Isle of Wight…I went and stayed in Portsmouth, so I was literally just a ferry ride across if Elizabeth couldn't cope with it…So it was just letting me put that little bit of, sort of like a safety net for Elizabeth, a bit of reassurance that I was there.

It seemed that parental presence provided a child-led and nurturing intervention, to best support Elizabeth’s specific needs. Sarah spoke further about the importance of interventions beyond a physical capacity, to a structural one:

She joined a lot of clubs…because it gave her more structure. So lunchtimes wasn't just a mass of girls hanging around…it was, “I've got this to do. This time, I've got to be in this, so I must have my lunch here and I must go there”…It just put structure through the whole day.

#### Fostering communication and relationships

As was evident, forming positive relationships with their child’s school was critical and whilst all talked about communications and relationships with the education system, parents had variable experiences. Allison had a positive experience of communication and support which spanned across her child’s primary and secondary schools:

When they started at secondary school, Adam had been put in a year group with nobody from his primary school at all. So I spoke to my head teacher at the primary school … and she said…‘You need to speak to SENCO at the secondary school’…I spoke to them and within a day, they’d taken Adam and said, ‘Right, which year group do you want to go in? Which one's got people in you know?’

The interaction between voicing concerns, and school staff hearing and responding seemed pivotal; the school’s rapid response provided a direct intervention supporting her child’s wellbeing. Similarly, Sarah spoke about support which occurred behind-the-scenes:

I had a couple of meetings with the SENCO…we discussed what Elizabeth’s needs would be, then she left it a couple of terms and then just emailed me and said, ‘Are you happy? Is Elizabeth happy? What's going on?’, and then the next year it was like ‘I haven't actually seen Elizabeth this year. She hasn't felt the need to come and see me. So I'm assuming everything's going fine.’

Earlier, established communication channels between school and parents seemed effective when Sarah’s child no longer needed to visit the school SENCO. Lydia viewed a good relationship as essential to ensure parents were not viewed negatively:

We have had some people ask us…‘You're always covered in bruises’. He's literally covered, you know, black and blue. Luckily, the school understands!

In contrast, Richard’s experience of maintaining good relations seemed difficult and frustrating:

I'm fed up of listening to them chortle with glee at how wonderful each other are…when I see them so obviously failing to perform their duties in the context of my child’s needs.

### Learning from covid-19 lockdown

Amidst this backdrop emerged a new issue, and all parents spoke about the ongoing COVID-19 pandemic and its impact on their child. It was through this experience that many parents discovered benefits of being at home with positive impacts on their child’s learning and wellbeing. Consequently, transitions back into school-based learning were met with apprehension.

#### Discovering the benefits of Home-Learning

Five parents spoke about benefits their child had experienced from home-learning compared to a school setting. Richard’s tone changed to positivity as he described how his child’s learning had progressed since home-learning began:

This whole working from home thing we've had during the lockdown, where Henry has been very, very good at following routines, and very good at keeping to his timetable and very good at completing online work…He's got the classroom-related distractions removed from him, he's actually making better progress than he would have done in the school environment. And that's incredible.

It appeared significant learning progress was attained in a home-learning environment, due to the removal of ‘classroom-related distractions,’ Lydia said:

We altered the timetable in the beginning to kind of suit how he was feeling. Didn't need the stuff that he had at school, which I thought was quite good progressions…Something that obviously we thought we were going to have to, because that's what he had at school.

Through the capacity to personalise her child’s timetable to ‘suit’ his moment-by-moment needs, Lydia seemed surprised to learn that completion of work was achieved without the aids required at school. Improved wellbeing of children throughout lockdown was discussed by Ryan through the simplification of social demands:

Sometimes when it comes to school, he'll be like, ‘I don't wanna go in today, because such and such was mean to me yesterday’, and it's almost a forceful battle to get him in. So it's quite nice that you've…not had that.

Clearly there was less scope for Ryan’s child to sense meanness within peer related social interactions at school, and the removal of ‘battle’ stressors during lockdown meant home-learning seemed beneficial for both parent and child.

Sarah touched on how home-learning had impacted on her child’s additional health needs by allowing more tailored pacing:

She gets the extra time, because of the slow thought processing and the hypermobility…She’s been able to work at her own pace, so if…she hasn't actually finished the task was set…she can run into her break if she wants to. I think managing her own time, within that given sort of school structure has really helped her.

Work itself did not seem to be the stressor, rather it was the constraints around when and how to do the work. Thus, Sarah’s child benefited from being in a home setting where she was able to work flexibly.

#### Transitions

With the transition out of home-learning back into school, four parents discussed their perception of their child’s wellbeing. Rachel stated:

It's just that change again, and I think after six weeks, it's hard. So after five months, it's going to be, you know, quite tricky.

Rachel reflected on how transitions were always tricky for her child, heightened after being away for an extended time. Allison echoed this idea, stating that:

He's not really left the house and I think it's gonna be quite tough for him transitioning back to some form of normality in September, that's gonna be really tough.

As a result of the COVID-19 pandemic, her child was having to negotiate repeated transitions, something which Allison perceived would be ‘really tough’. Sarah furthered this:

I do have the concerns that she, going back into school is going to be very overwhelming, because she's used to quietly sitting here doing her work…Everybody's going a bit hyper I think when they go back, and I’m anticipating there to be a few sort of little meltdowns …which Elizabeth to be fair, doesn't usually do it at school, she brings it home…’

## Discussion

The COVID-19 pandemic and consequent national lockdowns in the UK has meant most children have now experienced both school and home-learning providing a unique opportunity to explore the impact of a child’s ASD and/or health difficulties in both settings prior to the longer-term impact lockdown generated. All parents stated how the first COVID-19 lockdown and subsequent home-learning addressed the *mismatch of ASD features and school environments**^1^* by removing ASD and health related stressors, which subsequently, had a positive effect on their child’s wellbeing or learning progression. Indeed, when *discovering the benefits of home-learning* there was a sense that this enabled more AET (academic engaged time). These findings appear to confirm those reported anecdotally through clinical observations, suggesting some children with ASD prefer remote learning (Reicher, 2020).

While all parents spoke about benefits of home-learning, only one parent noted their child did well at school and at home. As several themes suggested home-learning fared better, given specific ASD and co-occurring health stressors were managed more flexibly; the impact of multiple issues on learning should be considered, particularly as additional health difficulties can often be overlooked in ASD (Joshi *et al.*, 2017). Differences in ASD presentations and the additional *impact of health issues* should also be considered, individualising the learning needs of children with ASD, especially as differences have been identified for those with the intellectual capacity to cope in mainstream education (de Giambattista *et al.*, 2019).

For some participants home-learning meant the removal of their child’s worry about bullying (Reicher, 2020). It seems pertinent some children struggled with adjustments at school, possibly due to fears of appearing different to their peers. Indeed, parents observed their child *masking* their *additional needs* or struggles through fear of peer and teacher views, and possible victimisation, in line with conceptualisations around masking in ASD (Pearson and Rose, 2021). It is also speculated that their differing emotional, health and social needs were better met at home, minimising misunderstandings from teachers and peers, allowing these children to flourish.

Within this study parents were clearly dedicated to caring for their children, yet also highlighted the stressful nature of having a child with ASD (Lai *et al.*, 2015); perhaps exacerbated for some by the battles to *access reasonable adjustments and support* in schools, alongside the necessity to *foster communication and relationships* with the school staff. However, parental stress appeared to diminish during home-learning in the first lockdown due to *parental learning interventions* matching their child’s learning with their moment-by-moment health and ASD related needs. With the opportunity to reduce sensory demands, provide positive, consistent nurturing relationships, and have greater flexibility around how and when to conduct learning, transferring these benefits into school settings could enhance the inclusivity agenda, which can be difficult to attain for ASD (Zhao *et al.*, 2021**)**. Indeed, what is critical for inclusion is not the place but rather a sense of belonging, fairness, feeling valued (by teachers), and support to access and thrive in education (Goodall, 2020), aspects that might already be more present for some within home environments. One way of attaining this might be via the promotion of ASD favourable environments (Bradley and Caldwell, 2013) and a satellite class transition model to facilitate inclusion within mainstream classrooms for young people who are able to manage this (Keane *et al.*, 2012), and flexibility in environments, tasks and routines for those that cannot (Lawrence, 2012). If a child is not managing in a school environment their wellbeing needs to be prioritised and supporting home-learning, even partially or temporarily, may be a helpful alternative option. This is important given generalisation in learning can occur between differing settings, people and activities (Carruthers *et al.*, 2020), potentially supporting the transition into adulthood for children with ASD and additional health needs.

The UK has repeatedly been advocating the re-establishment of all services including school-learning (Fegert *et al.*, 2020). Thus, for most young people, home-learning first ended in September 2020, before resuming in October, and again in January 2021. Young people have had to adapt to multiple transitions between school and home-settings; and any return to school-learning may present increased pressure for all children needing to catch-up. Indeed, whilst this transition may be difficult for young people in general (Darmody *et al.*, 2020) this study highlighted the extremity of the experience for young people with ASD (Makin *et al.*, 2017). With concerns around *transitions* back into school-learning, consideration should be given to *transitions* during unpredictable times to ensure the benefits accrued from inclusivity strategies and home-schooling are maintained. This might include flexibility based on the best interests of the individual child (Hurlbutt-Eastman, 2017), involving truly *reasonable adjustments* such as reducing subjects or full-time attendance requirements, beyond the usual consideration of small class environments or time out within lessons. As the results highlighted, *fostering* open and supportive *communications* between child, parents and educationalists may also be crucial to attain this.

It must be acknowledged the sample is small, limiting generalisability. Nevertheless, the sample size was appropriate for IPA especially as idiographic qualitative research aims to provide meaning to those with lived experience (Smith *et al.*, 2009). Additionally, due to this study focussing on child related background information, there are gaps in specific individual demographic information. Future research should explore the learning experiences for different ethnic or socio-economic groups, to identify if there are any disparities in experiences. Future research should also focus on how children with additional needs manage transitions back into school environments and how this may impact on their symptomologies and wellbeing over time. Finally, whilst home-learning may be beneficial for current well-being and academic attainment, future research should consider more systematic transitions which support children with ASD to navigate wider societal barriers as they enter adulthood, perhaps through the implementation of specific teaching protocols and the provision of specialist services.

## Conclusion

This study provides some understanding of the experiences of children with ASD and additional health difficulties in different learning environments, a previously unexplored area. The findings highlight how their complex needs were impacted by a mismatch with the school environment which seemed to be addressed when opportunities were provided for home-learning. These results hold real-world implications for the transition back into the school setting by transferring aspects within home-learning environments to enhance wellbeing and learning. These include genuine adjustments such as greater flexibility, nurturing interactions and reducing sensory demands. Adapting policies from the wider education system would help drive these initiatives. Standardising ASD friendly environments which also minimise sensory triggers would produce a norm minimising the potential of children with ASD standing out. For some children, even greater adaptations may be warranted, such as reductions in demands, flexible working or even supported home-schooling.

**Table 1.** Participant/Child Demographic Information.

Participant	Gender	Age	Marital status	Occupation	Age of child	Gender of child	Healthcare professional diagnoses/health difficulties	Age child diagnosed	Pre lockdown school absences due to diagnosis
Allison	Female	44	Married/Co-Habiting/Civil Partnership	Key Worker and Teaching Assistant	13	Male	Autism spectrum disorder	7	No
							Cerebral Palsy	2	Yes
							Generalised anxiety disorder	7	Yes
Lydia	Female	32	—^2^	Stay-at-home Parent	8	Male	Autism spectrum disorder	7	No
							Dyspraxia	7	No
							Hypermobility	5/6	No
							Anxiety	—^2^	No
Sarah	Female	55	Married/Co-Habiting/Civil Partnership	Self Employed and Retired	14	Female	Autism spectrum disorder	8	No
							Ehler Danlos Type 3 &	3	No
							Hypermobility		
Ryan	Male	—^2^	Married	Company Director	12	Male	Autism spectrum disorder	5	—^2^
							Attention deficit hyperactive disorder	5	Yes
Richard	Male	48	Married/Co-Habiting/Civil Partnership	Employed	15	Male	Autism spectrum disorder	6	No
							Attention deficit hyperactive disorder	6	Yes
							Low Self Esteem	—^2^	Yes
Rachel	Female	44	Married	Special Educational Needs Teacher	12	Male	Autism spectrum disorder	6	Yes
							Sensory Processing Disorder	—^2^	No
							Acute Anxiety^1^	—^2^	Yes

^1^Diagnosis not given by a Healthcare Professional (e.g. Doctor, Psychologist, Psychiatrist).

^2^A hyphen indicates the participant did not answer these questions when requested.

## Note

1 Themes from the results are shown in italics.
